# Investigation of Microstructure and Mechanical Properties of Extruded Mg–6Bi and Mg–6Bi–1Ag Alloys

**DOI:** 10.3390/ma17081853

**Published:** 2024-04-17

**Authors:** Xin Li, Jian Mao, Xuefei Huang, Weigang Huang

**Affiliations:** College of Material Science and Engineering, Sichuan University, Chengdu 610065, China

**Keywords:** dynamic recrystallization, dynamic precipitation, extruded Mg–Bi–Ag alloy, mechanical property, strain hardening

## Abstract

The extruded Mg–6Bi alloy and Mg–6Bi–1Ag alloy subjected to extrusion at 300 °C with the extrusion ratio of 25:1 and die-exit speed of 2 m/min were used to investigate microstructure characteristics and mechanical behavior. The experimental results demonstrate that the bimodal microstructure, composed of coarse dynamic unrecrystallized (unDRXed) grains and fine dynamic recrystallized (DRXed) grains, was obtained after extrusion. The Ag addition can obviously promote dynamic recrystallization and average grain size. It also indicates that the dynamic precipitation is significantly promoted by Ag addition during extrusion, obtaining more fraction of the Mg_3_Bi_2_ precipitates. Moreover, the extruded Mg–6Bi–1Ag alloy has a high tensile yield strength of 304 ± 2.0 MPa, which is increased by 19% compared to the extruded Mg–6Bi alloy, and elongation of 11.0 ± 1.7%, almost the same as 11.9 ± 0.9% of the extruded Mg–6Bi alloy. This result also shows that the extruded Mg–6Bi–1Ag alloy exhibits better strain hardening capacity. Therefore, Ag exhibits an effective role in promoting dynamic recrystallization and dynamic precipitation, resulting in the enhancement of strength and strain hardening capacity of the extruded Mg–6Bi–1Ag alloy, as well as keeping high ductility.

## 1. Introduction

It has been known that Magnesium and Magnesium alloys are some of the lightest metallic materials in the new century, which possess a significant potential value in the application field of automotive, aerospace, electronics, biomedical, and energy sectors because Mg alloys exhibit high specific strength and better biocompatibility [1]. To expand the engineering applications, some Mg alloys showing high strength and low cost have been researched and developed [2]. These alloys mainly include Mg–Zn-based alloys [3], Mg–Sn-based alloys [4], and Mg–Ca-based alloys [5,6]. In addition, the dynamic recrystallization and dynamic precipitation of Mg alloys could be promoted by the extrusion or rolling process, resulting in refined grain size and more content of precipitates. Consequently, these alloys can have high strength and ductility. Many works presented by researchers have indicated that Mg alloys with bimodal microstructure produced by the plastic deformation process exhibit high strength, accompanied by good ductility [4,7,8,9]. The fine grain size resulting from the dynamic recrystallization has a weak basal texture and easily leads to saturation of dislocation density within the fine grains with the tensile strain increasing. In contrast, the coarse dynamic unrecrystallized (unDRXed) grains with strong basal texture are prone to the storage of dislocation within grains with strain increasing, leading to high strain hardening. Therefore, the deformation compatibility at different deformation stages between fine dynamic recrystallized (DRXed) grains and large unDRXed grains can simultaneously improve the strength and ductility of Mg alloys.

Recently, Mg–Bi-based alloys have been found to exhibit good extrudability and high strength by an extrusion process or age hardening [10] due to the formation of Mg_3_Bi_2_ phases with a high melting temperature of 821 °C [11]. The Mg–Bi–Zn alloy reported by T.T. Sasaki [12] exhibits a great age-hardening property by aging treatment. The extruded Mg–0.3Bi alloy has an elongation of 170% at room temperature tensile test with a low strain rate, reported by Somekawa et al. [13]. Go et al. [11] indicated that the good extrudability and high tensile strength can be obtained for Mg–Bi–Al alloys under extrusion at 67 m/min die-exit speed. The work done by Meng et al. [14,15] illustrated that the extruded Mg–Bi–Ca alloy shows high tensile strength and good elongation of about 40% due to fine DRXed grain, and Mg_2_Bi_2_Ca and Mg_2_Ca precipitates in microstructure. Recently, Luo and Guo et al. [9] demonstrated that the extruded Mg–Bi–Sn–Mn and Mg–Sn–Bi–Mn alloys with bimodal microstructure present high strength and good ductility.

The Ag can improve the age-hardening ability of Mg alloys. Consequently, some Mg alloys with Ag, including Mg–Sn–Mn–Ag [16], Mg–Sn–Ag [17], Mg–Sn–Ca–Ag [18], Mg–Al–Sn–Ag [19], and Mg–Gd–Ag [20,21], have been investigated. The excellent age-hardening property of Ag-containing Mg alloys should mainly contribute to the accelerated formation of precipitates, increased density of precipitates, and fined precipitates [16,17,19,22]. In addition, it was found that Ag can segregate at the grain boundaries during rolling, bringing about the refinement of recrystallized grains by preventing grain growth [23]. Our previous work [24] has shown that Mg–6Bi–1Ag alloy illustrates a greater precipitation hardening response than Mg–6Bi alloy, which can be ascribed to the presence of refinement and increased area fraction of Mg_3_Bi_2_ precipitates.

Considering research into the extruded Mg–Bi–Ag alloy is hardly found at present, Mg–6Bi alloy and Mg–6Bi–1Ag alloy subjected to extrusion at 300 °C were used to study the relation of microstructure characteristics and mechanical behavior of the alloys. The influence of Ag on the dynamic recrystallization behavior, dynamic precipitation, and mechanical properties of Mg–6Bi–1Ag alloy after extrusion is analyzed in this work.

## 2. Materials and Methods

The raw materials of Mg (99.98%), Bi (99.99%), and Ag (99.99%) were used to prepare the alloy ingots. These pure raw metals were melted at 730 °C using an electric resistance furnace under a protective gas consisting of CO_2_ (99%) and SF_6_ (1%) mixture. The melt was stirred at certain intervals to ensure the chemical composition uniformly. After that, the melt was cast into a steel mold at 200 °C. The actual chemical compositions of the experiment alloys are 5.86 wt.% Bi of Mg-6Bi (MB) alloy and 6.05 wt.% Bi and 1.10 wt.% Ag of Mg–6Bi–1Ag (MBA) alloy, respectively, examined using an ICP instrument (inductively coupled plasma optical emission spectroscopy, ICP-OES, 5100 Agilent Technologies, Santa Clara, CA, USA). The samples for extrusion with 25 mm in diameter and 20 mm in length were cut from cast ingots. All samples for extrusion were treated for 10 h at 450 °C and then 2 h at 510 °C under cover using graphite powder to prevent oxidation. After that, the samples were quenched into room-temperature water. The extrusion for all samples was carried out at 300 °C with the die-exit speed of 2 m/min and extrusion ratio of 25:1. The diameter of extruded bars is 5 mm.

The phase structure and microstructure of the extruded samples were analyzed using optical microscopy (OM, MDS-400, Chongqing Optec Instrument Co., Ltd., Chongqing, China), scanning electron microscopy (SEM, JSM-6510LV, JEOL Ltd., Tokyo, Japan) and X-ray diffractometer (XRD, Smart Lab, Rigaku, Tokyo, Japan) with Cu-Kα radiation (λ = 1.54056 Å), scanning at a rate of 2°/min from 10° to 90° by 40 kV. The transmission electron microscopy equipped with Super-X EDS system and Cs probe corrector (TEM, FEI Titan Cubed Themis G2 300, FEI, Hillsboro, OR, USA) was used to determine the precipitates characteristics. The microstructure characteristics of the extruded samples were characterized by the electron backscattered diffraction (EBSD) method, including recrystallization behavior, texture, and misorientation of grains, etc. The EBSD data were obtained on an electron backscattered diffraction (EBSD) system (Velocity^TM^ Super, DEAX, Wolfforth, TX, USA) equipped with SEM (JSM-7200F, JEOL Ltd., Japan) and analyzed using AZtecCrystal software (https://nano.oxinst.com/azteccrystal, Oxford Instruments, Abingdon, UK). The size and content of the precipitates were determined using Image-Pro Plus 6.0 software (Media Cybernetics, Silver Spring, MD, USA) based on the TEM images.

The tensile specimens with round cross sections were obtained from extruded bars along the extrusion direction (ED), the gauge size of which is adopted as Ø2.5 mm × 25 mm by the standard ISO 6892-1:2019 [25]. The tensile test was carried out by the universal test machine (DDL-100, CIMACH Co., Ltd., Changchun, China) with 1 × 10^−3^ s^−1^ of strain rate at room temperature. Three tensile specimens were used for each test to obtain an average value of mechanical properties.

## 3. Results

### 3.1. Microstructural Characteristic after Solid Solution Treatment

**Figure 1:**
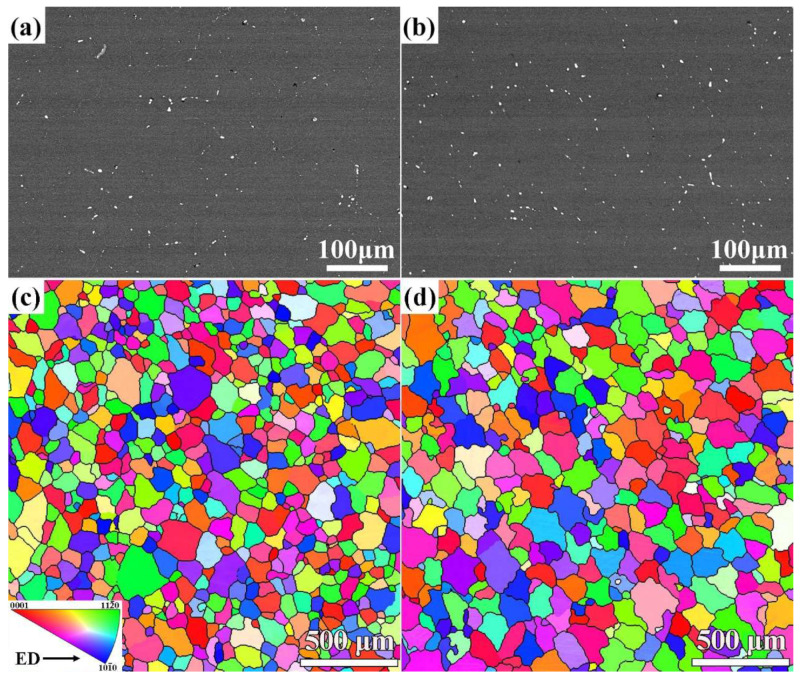
SEM images and inverse pole figures (IPF) of alloys subjected to solid solution treatment: (**a**,**c**) MB alloy, (**b**,**d**) MBA alloy.

Figure 1 reveals the SEM micrographs and inverse pole figures (IPF) based on EBSD analysis from a longitudinal section of MB alloy and MBA alloys subjected to solid solution treatment. It clearly shows that the undissolved secondary phases exist in two alloys. In terms of the composition of the alloys, these secondary phases should correspond to the Mg_3_Bi_2_ phases. As seen in Figure 1, the undissolved Mg_3_Bi_2_ phases mainly distribute at the grain boundaries (GBs). By the analysis with Image-Pro Plus 6.0 software, the area fractions of the undissolved Mg_3_Bi_2_ phases are 0.33% of solution-treated MB alloy and 0.35% of solution-treated MBA alloy. The average size of the Mg_3_Bi_2_ phase is 2.12 μm of solution-treated MB alloy and 2.55 μm of solution-treated MBA alloy. Although the size and content of the undissolved Mg_3_Bi_2_ phases are slightly larger for the solution-treated MBA alloy than those of the solution-treated MB alloy, there are no obvious differences between the two alloys subjected to solid solution heat treatment. As seen from Figure 1c,d, two alloys fully exhibit equiaxed grain after solid solution heat treatment by the present heat treatment process. The grain size is determined to be 79.8 ± 36.7 μm and 100.0 ± 44.4 μm respectively. Moreover, the grain orientation in two alloys indicates random distribution, that is no preferential orientation.

### 3.2. Microstructural Characteristics of the Alloys Subjected to Extrusion

**Figure 2:**
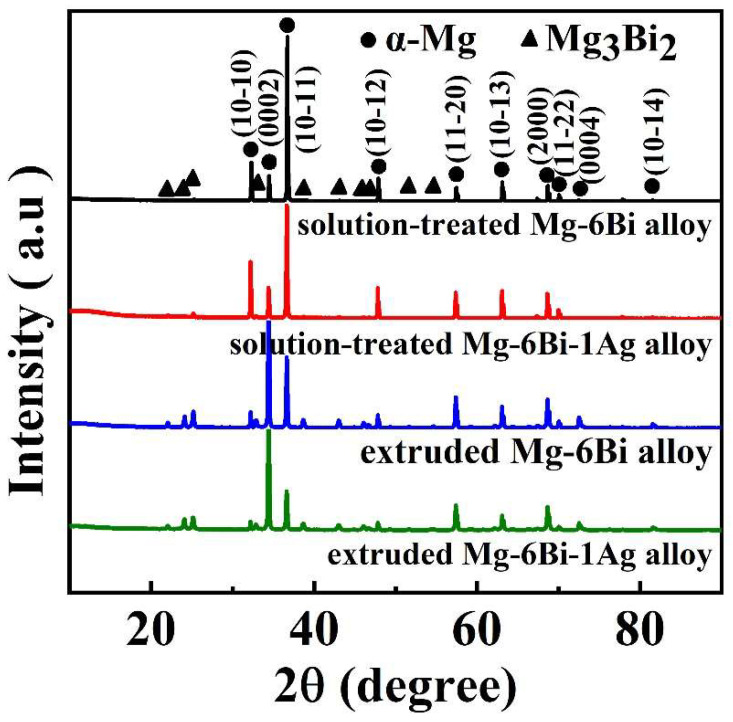
XRD results of the experiment alloys.

Figure 2 presents the XRD-measured results of the solution-treated and extruded two experiment alloys. It is seen that in addition to the α-Mg phase, a few of the Mg_3_Bi_2_ phase exists. According to the diffraction peak intensity of the Mg_3_Bi_2_ phase, Ag-containing alloy should be more undissolved than Mg_3_Bi_2_ precipitates after the same solid solution heat treatment. The XRD results are consistent with the results indicated in Figure 1. Moreover, the diffraction peaks of Mg_3_Bi_2_ phases in both as-extruded alloys exhibit stronger intensity than those of the solution-treated alloys. It means more Mg_3_Bi_2_ precipitates are present owing to the fact that extrusion deformation promotes secondary phase precipitation. In addition, the great diffraction peak intensities of (0002)_Mg_ of α-Mg matrix in two extruded alloys suggest that a large amount of (0002)_Mg_ in grains illustrates favorable orientation along ED. As a result, the basal texture may form in extruded alloys.

**Figure 3:**
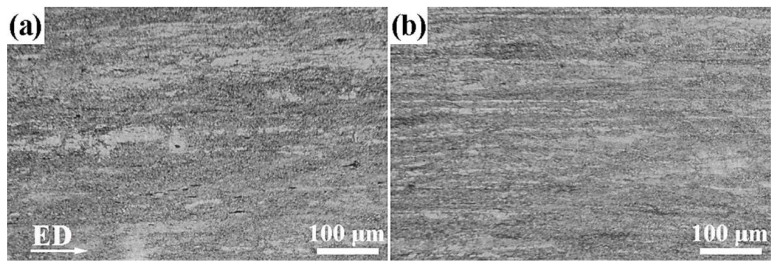
Optical micrographs of (**a**) extruded MB alloy and (**b**) extruded MBA alloy.

Figure 3 indicates the optical micrographs of extruded MB alloy and MBA alloy from longitudinal sections. It clearly illustrates that the unDRXed grains and DRXed grains in both extruded alloys obviously exhibit the direction along ED.

**Figure 4:**
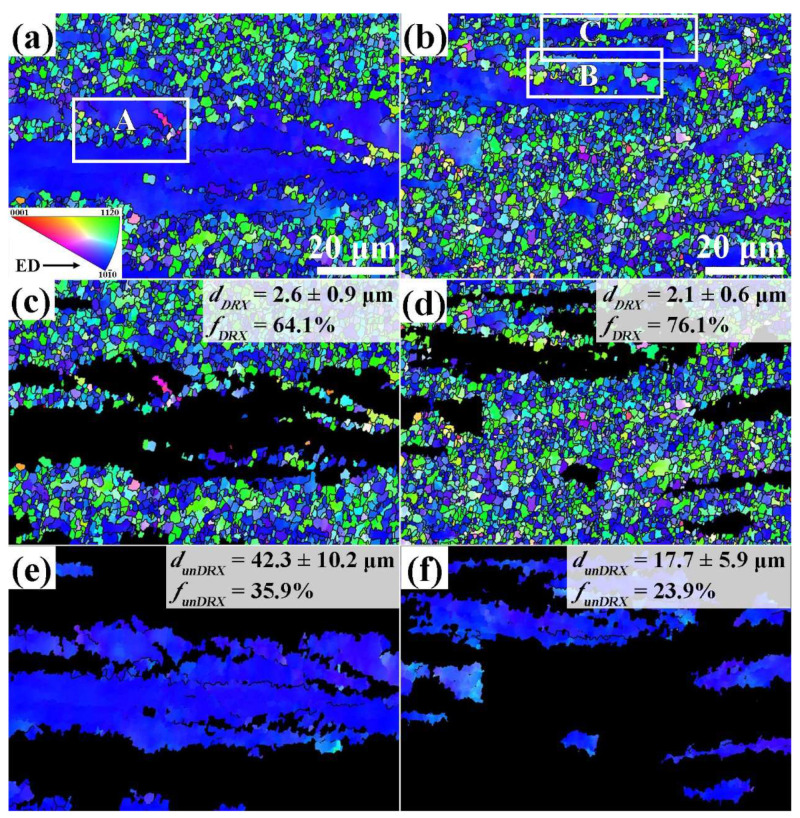
IPF maps of two extruded alloys: (**a**,**c**,**e**) whole grain region, DRXed grain region, unDRXed grain region for the extruded MB alloy, (**b**,**d**,**f**) for the extruded MBA alloy.

To characterize the microstructural characteristics of the extruded alloys, the EBSD analysis method was used. Figure 4 demonstrates inverse pole figure (IPF) maps of the extruded alloys. The IPF maps clearly exhibit that the microstructure of two extruded alloys is composed of the DRXed grains with fine size and coarse unDRXed grains elongated along ED. This kind of microstructure is commonly referred to as bimodal microstructure [26,27]. The average grain size and area fraction of DRXed grains are inserted in Figure 4. It is found that the area fraction of DRXed grains (*f_DRX_*) is 76.1% of the extruded MBA alloy and 64.1% of the extruded MB alloy. The average grain size of the DRXed grains (*d_DRX_*) is 2.1 ± 0.6 μm and 2.6 ± 0.9 μm of the extruded MBA and MB alloys, respectively. Meanwhile, for unDRXed grains, the extruded MBA alloy has 17.7 ± 5.9 μm average grain size, and the extruded MB alloy exhibits 42.3 ± 10.2 μm average grain size. Therefore, it clearly illustrates that Ag addition can promote the dynamic recrystallization behavior of MBA alloy during extrusion, resulting in an increase in the content of the DRXed grains. The results also show that the unDRXed grain size is significantly refined for Ag-containing alloy during the extrusion process, which should be due to the content reduction of the unDRXed grains. As indicated in Figure 4, the DRXed grains in two extruded alloys mainly exhibit orientations corresponding to <10$\overset{-}{1}$0>//ED and <11$\overset{-}{2}$0>//ED. All unDRXed grains present the same <10$\overset{-}{1}$0>//ED orientation.

### 3.3. Texture in the Extruded Alloys

**Figure 5:**
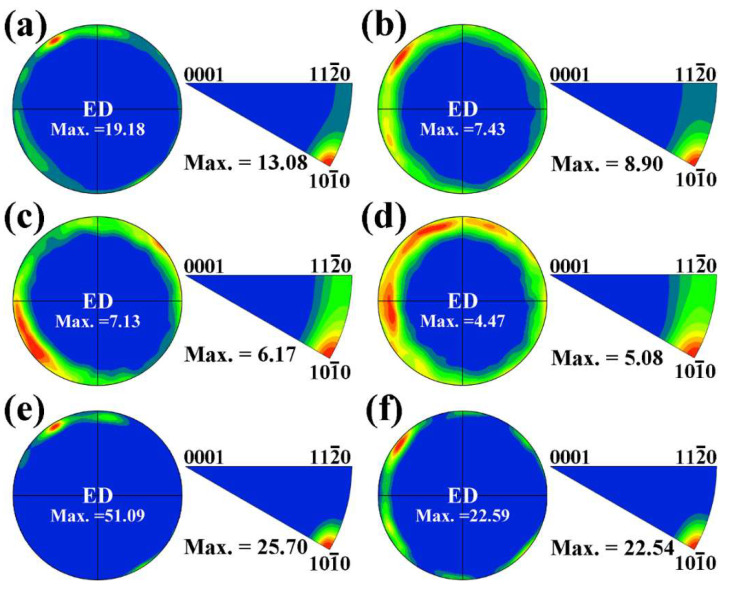
(0001) PFs and ED IPFs in whole grain region, DRXed grain region, and unDRXed grain region: (**a**,**c**,**e**) for the extruded MB alloy, (**b**,**d**,**f**) for the extruded MBA alloy.

Figure 5 provides (0001) pole figures (PFs) and IPFs along ED for the whole grain region, DRXed grain region and unDRXed grain region of two extruded alloys. As illustrated in Figure 5a,b, two extruded alloys have some basal fiber texture, which exhibits (0001) planes, <10$\overset{-}{1}$0> and <11$\overset{-}{2}$0> crystalline direction parallel to ED. The extruded MB alloy appears the maximum texture intensities of 19.18 in (0001) PF, and 13.08 in ED IPF, whereas the extruded MBA alloy has maximum texture intensities of 7.43 and 8.90, respectively. This demonstrates that the texture intensity in the extruded MB alloy is stronger than that of the extruded MBA alloy. Furthermore, the <10$\overset{-}{1}$0> fiber texture component reveals stronger intensity than that of the <11$\overset{-}{2}$0> fiber texture component. Figure 5c–f exhibit the (0001) PFs and ED IPFs of the DRXed grain region and unDRXed grain region in the two extruded alloys. As seen in Figure 5c,d, the maximum texture intensities of the DRXed grain region in the extruded MB alloy are 7.13 in the (0001) PF and 6.17 in ED IPF. The maximum texture intensities are higher than those in the extruded MBA alloy, which are 4.47 and 5.08, respectively. Two extruded alloys indicate the stronger <10$\overset{-}{1}$0> fiber texture and weaker <11$\overset{-}{2}$0> fiber texture components in the DRXed grain regions. As indicated in Figure 5e,f, the unDRXed grain regions of both alloys only reveal the <10$\overset{-}{1}$0> basal fiber texture. The unDRXed grain region of the extruded MB alloy illustrates the maximum texture intensities of 51.09 in the (0001) PF and 25.70 in ED IPF, while as for the extruded MBA alloy, the maximum texture intensities are 22.59 in the (0001) PF and 22.54 in ED IPF. This clearly demonstrates that the DRXed grains have two fiber texture components: the stronger <10$\overset{-}{1}$0> texture and weaker <11$\overset{-}{2}$0> texture, while the unDRXed grains only show <10$\overset{-}{1}$0> fiber texture component, which indicates that the <10$\overset{-}{1}$0> direction in unDRXed grains should be rotated to parallel to extrusion direction during extrusion. Therefore, the dynamic recrystallization that occurs during extrusion can weaken the basal texture in extruded alloys.

**Figure 6:**
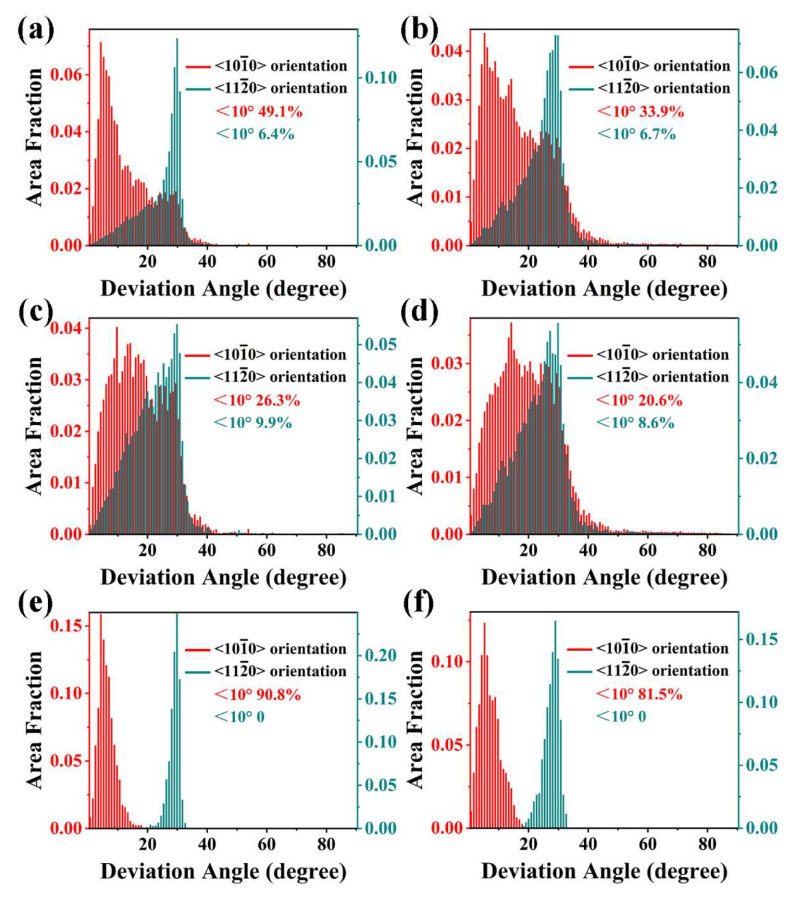
Deviation angles of <10$\overset{-}{1}$0> and <11$\overset{-}{2}$0> orientation from ED in whole grain region, DRXed grain region, and unDRXed grain region: (**a**,**c**,**e**) for the extruded MB alloy, (**b**,**d**,**f**) for the extruded MBA alloy.

To further analyze the area fraction of the texture components of <10$\overset{-}{1}$0>//ED and <11$\overset{-}{2}$0>//ED, the distributions of deviation angles of <10$\overset{-}{1}$0> and <11$\overset{-}{2}$0> orientations with ED were determined from EBSD results, as indicated in Figure 6. The deviation angles of both <10$\overset{-}{1}$0> and <11$\overset{-}{2}$0> orientations for most grains in the extruded alloys are less than 40° from ED. Here, the <10$\overset{-}{1}$0> and <11$\overset{-}{2}$0> orientations parallel to ED with the deviation angles within 10° are taken for the <10$\overset{-}{1}$0> and <11$\overset{-}{2}$0> basal fiber texture components. Therefore, the area fraction of <10$\overset{-}{1}$0> and <11$\overset{-}{2}$0> basal fiber texture components can be obtained.

The results presented in Figure 6 demonstrate that the whole area fraction of <10$\overset{-}{1}$0> basal fiber texture components is 49.1% in the extruded MB alloy and 33.9% of the extruded MBA alloy, while the <11$\overset{-}{2}$0> basal fiber texture components are 6.4% and 6.7% respectively. The results show that Ag can reduce the area fraction of <10$\overset{-}{1}$0> basal fiber texture in extruded MBA alloy, which can be ascribed to Ag promoting the dynamic recrystallization, leading to an increase of DRXed grains. The area fraction of the <10$\overset{-}{1}$0> basal fiber texture component in the DRXed grains is 26.3% of extruded MB alloy and 20.6% of extruded MBA alloy, while the <11$\overset{-}{2}$0> basal fiber texture components are 9.9% and 8.6%, respectively. This means that more fractions of the <10$\overset{-}{1}$0> basal fiber texture exist in the DRXed grains of both extruded alloys than <11$\overset{-}{2}$0> basal fiber texture. Figure 6e,f indicate that only <10$\overset{-}{1}$0> basal fiber texture component is present in the unDRXed grains, the area fraction of which is 90.8% of the extruded MB alloy and 81.5% of the extruded MBA alloy, respectively.

### 3.4. Characteristics of Dynamic Precipitates

**Figure 7:**
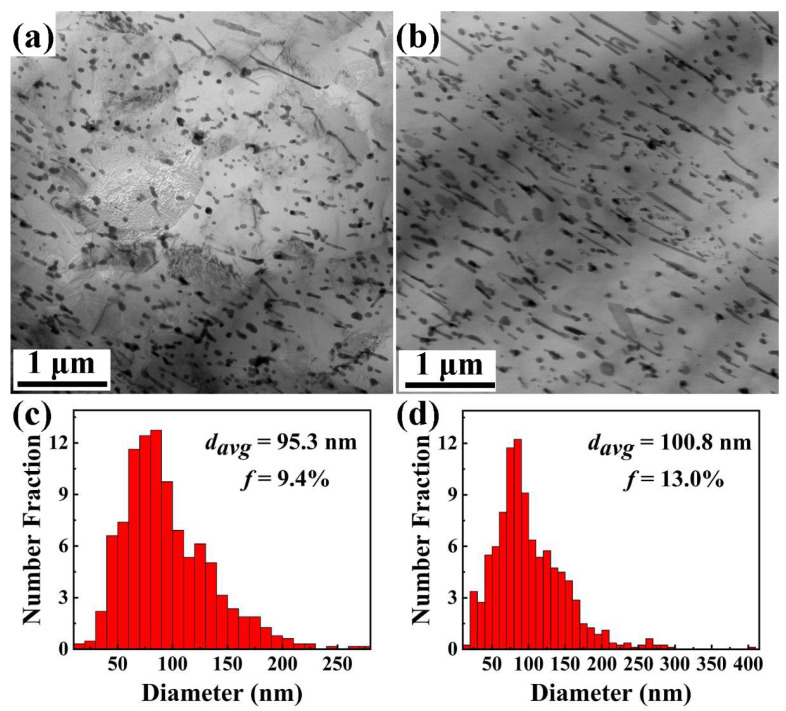
Bright-field TEM images: (**a**) extruded MB alloy, (**b**) extruded MBA alloy. Statistics of the average diameter of dynamic precipitates: (**c**) extruded MB alloy, (**d**) extruded MBA alloy.

The TEM images of dynamic precipitates in both extruded alloys and the precipitate size distribution are given in Figure 7. Compared to the alloys treated by solid solution quenching, there are many nanosized precipitates in both extruded alloys, which should correspond to the dynamic precipitation behavior during the extrusion deformation. As seen in Figure 7, the dynamic precipitates exhibit the morphologies of granular, rod, and lath shape, and are distributed within the grains and at the grain boundaries of the Mg matrix. The results in Figure 7c,d show that the area fraction of the dynamic precipitates is 9.4% in the extruded MB alloy and 13.0% in the extruded MBA alloy. Thus, this clearly indicates that the dynamic precipitation behavior of precipitates can be promoted through the Ag addition, leading to the increase of the precipitate contents. However, it is found that the average size of the precipitates in extruded MBA alloy is 100.8 nm and 95.3 nm in extruded MB alloy. In addition, the extruded MBA alloy has more lath-shaped precipitates than the extruded MB alloy. As reported by Weng et al. [28], the lath-shaped precipitates have an aspect ratio greater than 2. In the present work, the average length and width of lath-shaped precipitates is 221 nm and 65 nm in extruded MB alloy, and 247 nm and 63 nm in extruded MBA alloy. The width of the lath-shaped precipitates is almost the same for the two extruded alloys, whereas the average length of precipitates in the MBA alloy is longer than that in the extruded MB alloy. Therefore, the fact that the extruded MBA alloy has a slightly large average size of the precipitates can be ascribed to the lath-shaped precipitates showing the longer length and larger area fraction.

**Figure 8:**
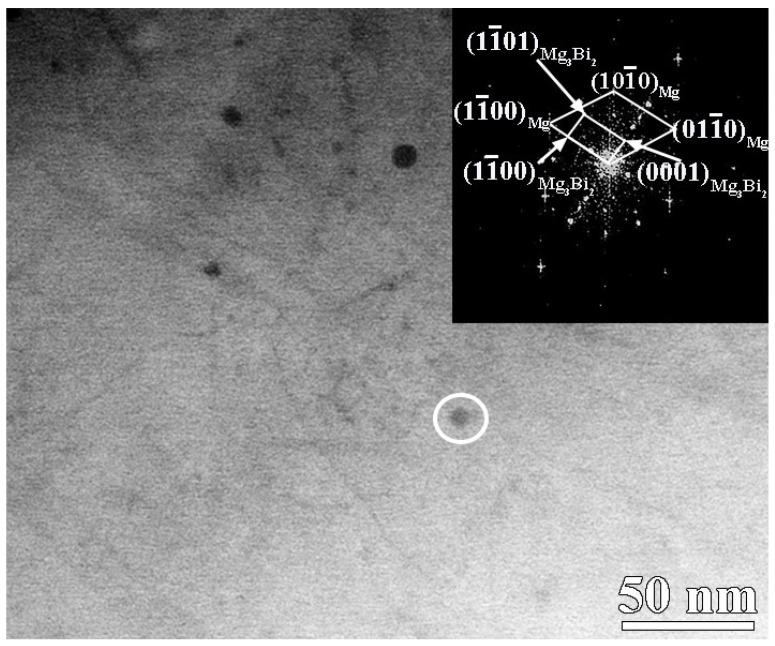
The TEM images of nanosized precipitates and dislocations in extruded MBA alloy.

It is worth noting that the nanosized precipitates with a diameter of about 7 nm are formed within the grains of the extruded MBA alloy during extrusion, as indicated in Figure 8. It is also seen that many dislocations exist near the nanosized precipitates. These nanosized precipitates can be analyzed by the fast Fourier transform (FFT) pattern to be the Mg_3_Bi_2_ phase, as seen from a representative precipitate circled by a white line in Figure 8. The nanosized Mg_3_Bi_2_ phase and α-Mg matrix can hold [11 $\overset{-}{2}$ 0]_Mg3Bi2_//[0001]_Mg_, (0001)_Mg3Bi2_//(11$\overset{-}{2}$0)_Mg_ and (1$\overset{-}{1}$00)_Mg3Bi2_//(1$\overset{-}{1}$00)_Mg_ orientation relationships (ORs), which is in accordance with the OR1 in our previous work [24] and the result from Sun, et al. [28]. However, these kinds of nanosized precipitates are not found in the extruded MB alloy. This result further demonstrates that Ag atoms, as well as dislocation, have a great effect on accelerating the formation of the dynamic precipitates of the extruded MBA alloy. As is well known, the dislocation density in the Mg matrix increases due to interaction between dislocation moving and grain boundaries or second particles during extrusion, which can increase the diffusion of Ag atoms. As a result, Ag clusters or Ag–Bi clusters can be formed easily [24]. The presence of these clusters in the Mg matrix can reduce the nucleation energy of precipitates, resulting in an increase in the volume fraction of dynamic precipitates. Furthermore, the high density of dislocation and tangles of dislocations also can provide many nucleation sites of dynamic precipitates, promoting the formation of dynamic precipitates [29], which is consistent with the results indicated in Figure 8. Thereby, the extruded Mg–6Bi–Ag alloy exhibits a larger amount of Mg_3_Bi_2_ precipitates than that of the extruded Mg–6Bi alloy.

### 3.5. Mechanical Properties

**Figure 9:**
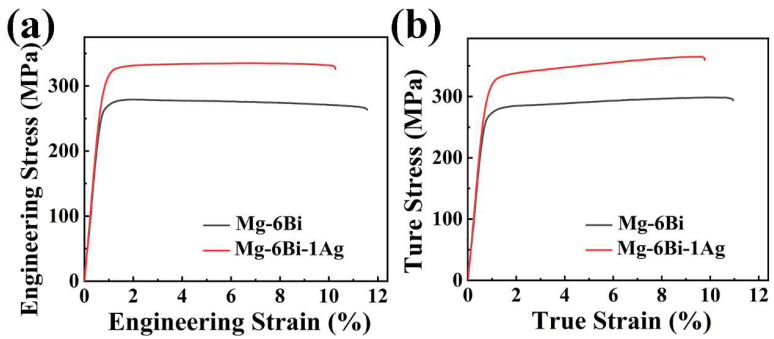
Tensile stress-strain curves of both extruded alloys: (**a**) engineering stress-strain curves, (**b**) true stress-strain curves.

Figure 9 illustrates the engineering stress-strain and true stress-strain cures of both extruded alloys by tensile test. The values of mechanical properties obtained from Figure 9a,b are given in Table 1. As seen from Figure 9, the experiment alloys display a good uniform deformation behavior with the strain increasing. In Table 1, the extruded MBA alloy possesses the ultimate tensile strength (*σ*_UTS_) of 332 ± 4.8 MPa and yield strength (*σ*_0.2_) of 304 ± 2.0 MPa. The *σ*_UTS_ and *σ*_0.2_ of the extruded MB alloy are increased by 56 MPa and 48 MPa, respectively, owing to Ag addition. As illustrated in Table 1, the tensile elongation is 11.9 ± 0.9% of the extruded MB alloy and 11.0 ± 1.7% of the extruded MBA alloy. It suggests that the large average size and high area fraction of the precipitates in the extruded MBA alloy may cause the elongation to be slightly low. These large-size precipitates can inhibit the dislocation slip during the plasticity deformation, thus easily causing the stress concentration and cracks forming at the interfaces between precipitates and the α-Mg matrix [30].

**Figure 10:**
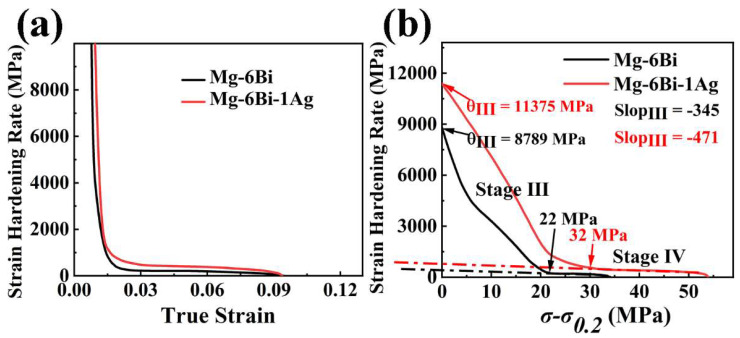
The strain hardening rate curves of two extruded alloys: (**a**) strain hardening rate vs. true strain, (**b**) strain hardening rate vs. (*σ-σ*_0.2_).

Figure 10a presents the strain hardening rate versus true strain curves of the extruded alloys. The strain hardening rate (*θ*) is expressed as [31]:

(1) $$\theta =\frac{d\sigma }{d\varepsilon }$$

where *σ* and *ε* mean tensile true stress and true stain, respectively. It is seen from Figure 10a that the strain hardening rate of both extruded alloys rapidly decreases at the initial stage of deformation as the strain increasing because of a short elastoplastic transition. Then, the strain hardening rate decline slowly and linearly with the strain increasing. The high strain hardening rate at all plastic deformation stages is observed in the extruded MBA alloy, which mean the higher strain hardening capacity for the extruded MBA alloy than that for the extruded MB alloy. The strain hardening capacity ($H_{c}$) of alloys can be defined as follows [32,33]:

(2) $$H_{c}=\frac{\sigma_{UTS}-\sigma_{0.2}\;}{\sigma_{0.2}}$$

where *σ_UTS_* is true ultimate tensile strength, *σ*_0.2_ express true yield strength (true 0.2% proof stress). Thus, the *Hc* value of the extruded MB and MBA alloys, obtained from Equation (2), are 0.14 and 0.18, respectively. It indicates that the strain hardening capacity of the extruded MB alloy can improved by Ag alloying.

The uniform plastic deformation behavior of metallic materials is described by following the Hollomon equation [32,34]:

(3) $$\sigma =K\varepsilon^{n}$$

in Equation (3), *σ* expresses the tensile true stress, *K* is the strength coefficient, *ε* means the tensile true strain, *n* indicates the strain hardening exponent. According to the Equation (3), *n* can be expressed as follows:

(4) $$n=\frac{dln\sigma }{dln\varepsilon }$$

Therefore, based on Equation (4), *n* values can be obtained by fitting the *lnσ-lnε* curves. The results indicate that average *n* value is 0.0568 for the extruded MBA alloy and 0.0308 for the extruded MB alloy. It is clearly seen that Ag alloying is favorable to the uniform plastic deformation and better strain hardening capacity of the extruded MBA alloy.

To further analyze the strain hardening behavior of the extruded alloys after yield stress, the *θ* vs. (*σ-σ*_0.2_) curves, where (*σ-σ*_0.2_) represents net flow stress, *σ*_0.2_ is the true 0.2 proof stress, are plotted, as exhibited in Figure 10b. It is seen that the *θ* of two alloys firstly decreases linearly with the (*σ-σ*_0.2_) increasing, showing the strain hardening behavior described as stage III, which was designated by Kocks and Mecking in fcc polycrystals [31]. This stage is related to the dynamic recovery (DRV) [31,35,36,37,38]. Then *θ* becomes slowly decreased with the increase in net flow stress (*σ-σ*_0.2_), which is noted as stage IV at larger net flow stress [31,35,36]. Stage IV is related to the dislocation rearrangements or formation of cell wall substructure [31,38,39]. The net flow stress at *θ* transition from stage III to stage IV is about 32 MPa for MBA alloy and 22 MPa for MB alloy. The θ_III_ value expressing the strain hardening value at stage III can be obtained by extrapolating the curves of stage III to (*σ-σ*_0.2_) *=* 0 in Figure 10b. The results illustrate that extruded MBA alloy has a higher θ_III_ value compared to the extruded MB alloy, indicating a high strain hardening effect at stage III of deformation. The slope of the curve at stage III connects with dynamic recovery during tensile deformation. The larger slope value of the curve at stage III for the extruded MBA alloy implies a greater dynamic recovery rate, as indicated in Figure 10b. Furthermore, Ag can make stage IV deformation of the extruded MBA alloy longer, which means the deformation of the sample continues for a longer time due to the high strain hardening capacity. As reported by T. Ungár [39], this behavior is related to the substructure (cell block) size of dislocations.

## 4. Discussion

### 4.1. Influence of Ag on the DRX Behavior

In terms of present results, it can be determined that Ag addition has a favorable role in promoting the DRX of the extruded MBA alloy during extrusion at 300 °C, increasing the content of the DRXed grains. To further comprehend the role of Ag on DRX in MB alloy, the DRX behaviors of two alloys are analyzed as follows.

**Figure 11:**
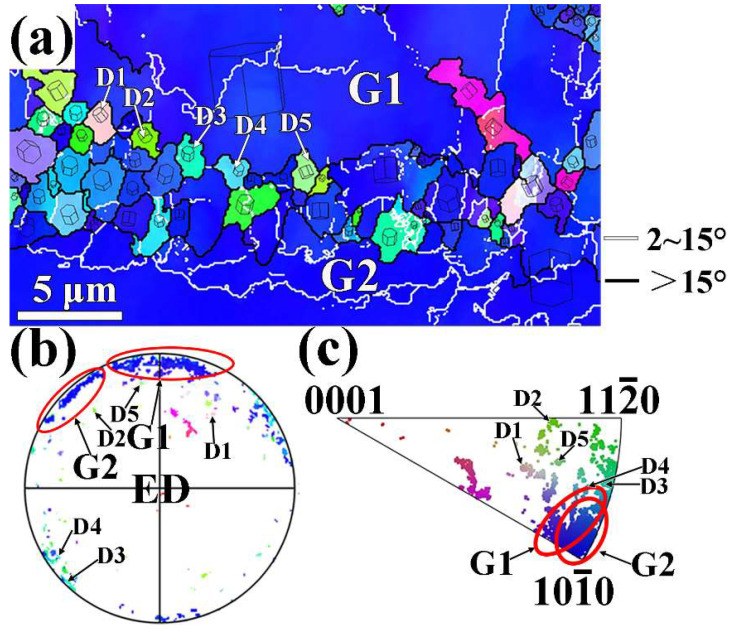
EBSD analysis of region A selected from Figure 4a: (**a**) IPF map, (**b**) (0001) PF, (**c**) ED IPF.

Figure 11a presents the enlarged IPF map of region A selected in Figure 4a for the extruded MB alloy. According to the results shown in Figure 11b,c the G1 and G2 grains are two unDRXed grains corresponding to <10-$\overset{-}{1}$0> basal fiber texture. The DRX has occurred during extrusion on the elongated original grain boundary with high angle misorientation between the G1 and G2 unDRXed grains, forming the fine DRXed grains. Based on the results illustrated in Figure 11b,c, the DRXed grains D1, D2, D3, D4, and D5 formed near the original high-angle boundaries (HAGBs) exhibit different orientations from the G1 and G2 parent grains and randomized orientation compared to unDXRed grain (G1 and G2) with <10-$\overset{-}{1}$0> fiber orientation. Consequently, these DRXed grains formed at original grain boundaries should be produced by discontinuous dynamic recrystallization (DDRX) mechanism during extrusion [40]. Furthermore, the bulging of original high-angle boundaries (HAGBs) toward unDRXed grain G2 is clearly observed, as indicated in Figure 11a. Although some sub-grains and low-angle grain boundaries (LAGBs) are observed within the unDRXed grain (G1 and G2), the DRXed grains transformed from these LAGBs and sub-grains by continuous dynamic recrystallization (CDRX) mechanism are seldomly found. It suggests that the DRXed grain is difficult to form within the unDRXed grain by the CDRX mechanism after the completion of extrusion.

**Figure 12:**
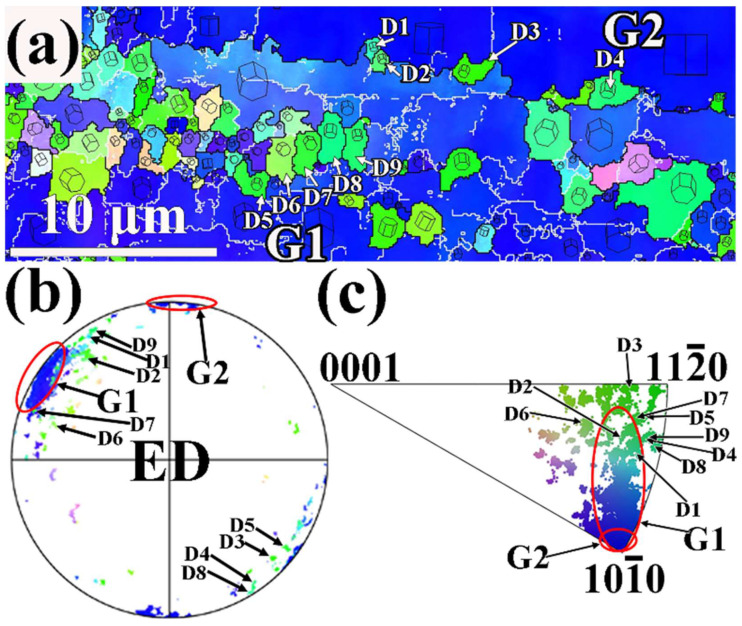
EBSD analysis results of region B in Figure 4b: (**a**) IPF map, (**b**) (0001) PF, (**c**) ED IPF.

Figure 12 illustrates the enlarged IPF map of region B selected in Figure 4b, as well as related (0001) PF and ED IPF for extruded MBA alloy. Region B exhibits two unDRXed grains (G1 and G2) with different grain orientations, as described in Figure 12b. Compared to MB alloy, a few of DRXed grains are present at the high-angle grain boundaries between G1 and G2 grains. Figure 12b indicates that the orientation of the fine D1, D2, D3, and D4 DRXed grains near the grain boundaries is close to the orientation of the unDRXed grain G1. Nevertheless, it is seen from Figure 12c, that D1, D2, D3, and D4 DRXed grains exhibit differences from the <10$\overset{-}{1}$0>//ED orientation of unDRXed G1 and G2 grains. Among these DRXed grains, D3 grain is close to <11$\overset{-}{2}$0>//ED, whereas D1, D2, and D4 grains shows the orientation between <10$\overset{-}{1}$0> and <11$\overset{-}{2}$0> parallel to ED. In terms of the analysis above, it can be suggested that these DRXed grains are formed by CDRX mechanisms [40,41]. In general, the grain boundaries are the preferred sites of DRXed grain nucleation during DDRX. However, in region B, the DDRX behavior hardly occurs at the grain boundary between unDRXed grain G1 and G2. The reason should be due to more undissolved Mg_3_Bi_2_ particles located on the grain boundary of the extruded MBA alloy, resulting in reducing the mobility of grain boundaries by the grain boundary pining effect of particles. As a result, DDRX behavior is inhibited [42,43].

Many research works [7,43,44] indicate that particles with a size of more than 1 μm can expedite dynamic recrystallization by particle-stimulated nucleation (PSN) mechanism. Although more undissolved Mg_3_Bi_2_ particles with a size of more than 2 μm lay on the grain boundaries in the present experiment, the DRX does not occur by PSN mechanism. This result indicates that the retarding effect on grain boundary migration by secondary particles pining grain boundaries is dominant, leading to inhibiting DRX. Moreover, it is worthing to note that many fine DRXed grains form within G1, as illustrated in Figure 12a. Besides, a lot of sub-grains and LAGBs are also observed within the G1 unDRXed grain. From Figure 12b,c the crystallographic orientation of DRXed grains D5-D9 is close to the crystallographic orientation of the unDRXed grain G1. These DRXed grains exhibit the orientation between <10$\overset{-}{1}$0> and <11$\overset{-}{2}$0>, even closer to <11$\overset{-}{2}$0> parallel to ED. Therefore, these DRXed grains should form by the CDRX mechanism [40,41]. This demonstrates that Ag can promote the dynamic recrystallization of the extruded MB alloy by the CDRX mechanism.

**Figure 13:**
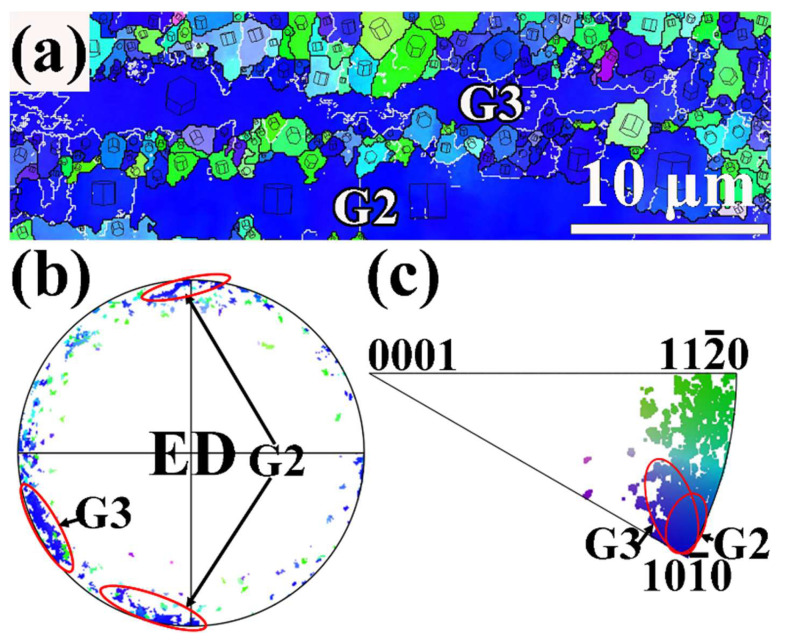
EBSD analysis results of region C in Figure 4b: (**a**) IPF map, (**b**) (0001) PF, (**c**) ED IPF.

Figure 13 presents the enlarged IPF map of the extruded MBA alloy in region C marked from Figure 4b, and related (0001) PF and ED IPF. According to the result indicated in Figure 13b,c, the G3 grain is another unDRXed grain, which exhibits a different orientation from the G2 unDRXed grain. Many fine DRXed grains form at the grain boundaries between G2 and G3 by DDRX mechanism. Therefore, the DRX behaviors for the extruded MBA alloy cover the DDRX and CDRX mechanism, and the CDRX behavior is dominant. Thereby, the content of DRXed grains increases after extrusion at 300 °C.

In the present study, although the initial grain size (83.8 μm) of a solution-treated MBA alloy is slightly larger than that (74.3 μm) of a solution-treated MB alloy, no obvious difference between the two values can be regarded to influence the DRX behavior. Because the deformation twins are not found in both extruded alloys, as the results of Mg–Bi alloy indicated by Go et al. [43], the influence of twins on the DRX can be ignored. However, the dynamic precipitation behavior in the two experiment alloys appears obviously different during the extrusion. The Ag addition promotes the dynamic precipitation for MBA alloy, leading to the increase of Mg_3_Bi_2_ precipitates. From the results in Figure 7, the average size of dynamic Mg_3_Bi_2_ precipitates is also no significant difference, which is about 95.3 nm of extruded MB alloy and 100.8 nm of extruded MBA alloy. However, the extruded MBA alloy has more content of dynamic precipitates than the extruded MB alloy. And some nanosized Mg_3_Bi_2_ precipitates with about 7 nm form within grains. Therefore, it can be inferred that the influence of Ag on the dynamic recrystallization of MBA alloy during extrusion should be related to dynamic precipitates.

The secondary phase particles illustrate a double-edged effect on the recrystallization [7,43,45,46]. The larger particles with a size of more than 1 µm can accelerate the DRX by PSN mechanisms. The fine secondary phase particles inhibit the grain boundary migration by Zener pining effect [45], leading to retardation of DRX during extrusion, especially for nucleation of DRXed grains by the DDRX mechanism. As for the effect of fine particles on the DRX, Shen et al. [47] and Zou et al. [44] indicated that nanosized precipitates can accelerate the CDRX and cause the refinement of the DRXed grains. The present results also indicate that more nanosized Mg_3_Bi_2_ precipitates form in extruded MBA alloy, which promote CDRX. Thus, area fraction of the DRXed grains increase and the fine DRXed grains are obtained. This result can be understood by the following reasons. The retained high dislocation density can be obtained due to the pining dislocation by nanosized precipitates, which can retard the dynamic recovery (DRV), resulting in enhancement of the driving force for CDRX [47]. For the other reason, the fine precipitates can provide a pinning force to impede grain boundary migrating and the grain growth, resulting in refinement of DRXed grains.

### 4.2. Strengthening Mechanism

The present results demonstrate that Ag can accelerate dynamic recrystallization and dynamic precipitation of the extruded MBA alloy. As a result, MBA alloy reveals the refined DRXed grains and high strength. The main reasons can be attributed as follows.

#### 4.2.1. Grain Boundary Strengthening

As known from microstructures in study, two extruded alloys exhibit the bimodal microstructure composed of fine DRXed grains and large unDRXed grains. The yield strength of the bimodal microstructure can be estimated using the modified Hall–Petch equation [9,48,49,50,51]:

(5) $$∆\sigma_{gb}={f_{DRX}k}_{DRX}{d_{DRX}}^{-1/2}+{f_{unDRX}k}_{unDRX}{d_{unDRX}}^{-1/2}$$

where the *f_DRX_* and *f_unDRX_* express the area fraction of the dynamic recrystallized grains and undynamic recrystallized grains, the *k_DRX_* and *k_unDRX_* indicate the Hall–Petch constant, the *d_DRX_* and *d_unDRX_* indicate the average grain size of the dynamic recrystallized grains and undynamic recrystallized grains. In the present work, DRXed grains and unDRXed grains illustrate different texture intensity. Thus, the value of *k_DRX_* is 188 MPa·μm^1/2^ and *k_unDRX_* is 303 MPa·μm^1/2^ [48,50]. The *f_DRX_* and *f_unDRX_* are 64.1% and 35.9% of the extruded MB alloy, and 76.1% and 23.9% of the extruded MBA alloy. *d_DRX_* and *d_unDRX_* are 2.6 μm and 42.3 μm of the extruded MB alloy, while *d_DRX_* and *d_unDRX_* are 2.1 μm and 17.7 μm of the extruded MBA alloy. Therefore, the increment of strength resulted from grain boundary strengthening is 92 MPa of extruded MB alloy, and 116 MPa of extruded MBA alloys.

#### 4.2.2. Dislocation Strengthening

As is well known, the basal dislocation and non-basal dislocation can be activated during extrusion. Thus, although many dislocations will be eliminated by dynamic recrystallization, the residual dislocations can remain within the grains and near the grain boundaries of alloys by pinning effect from fine precipitates and hindering dislocation moving at grain boundaries after extrusion. Most of these residual dislocations exist as a form of low-angle grain boundary and sub-grains, which could be considered as the geometrically necessary dislocation (GND) [52,53]. Consequently, these residual dislocations should provide the strengthening contribution to the alloys. The strength increasement came from the dislocations can be determined from the following equation [54]:

(6) $${∆\sigma }_{d}=M\alpha Gb\sqrt{\rho^{GND}}$$

in the equation, *M* expresses Taylar factor (2.5), α indicates a constant of 0.2, *b* express the Burgers vector of Mg (0.32 nm) [55], *G* means shear modulus of Mg (1.66 × 10^4^ MPa) [56], and *ρ* expresses the GND density, which is 8.8 × 10^14^ m^−2^ of the extruded MB alloy and 10.7 × 10^14^ m^−2^ of the extruded MBA alloy, obtained from the EBSD. Consequently, the Δ*σ_d_* is estimated as 79 MPa of the extruded MB alloy and 87 MPa of the extruded MBA alloy.

#### 4.2.3. Precipitation Strengthening

As illustrated in present result, the dynamic precipitation occurs in two alloys during the extrusion process and many fine precipitates are obtained. These precipitates can strengthen the alloys by hindering dislocation moving. The increment of strength could be calculated by Orowan strengthening mechanisms [57]. Considering the basal slip as a dominant active system during room temperature tensile deformation, the increment of strength produced by fine Mg_3_Bi_2_ precipitates can be analyzed using the following equation presented by Nie et al. [57,58,59].

(7) $$∆\sigma_{p}=M\frac{Gb}{2\pi \sqrt{1-\nu }\;\left(\frac{0.953}{\sqrt{f}}-1\right)d_{p}}\ln\frac{d_{p}}{b}$$

where *M* is the Taylar factor (2.5), *b* means the Burgers vector of Mg (0.32 nm), *G* indicates the shear modulus (1.66 × 10^4^ MPa for Mg), *d_p_* expresses the average size of the Mg_3_Bi_2_ precipitates, *ν* means the Poisson’s ratio (0.33 for Mg) [56], and *f* indicates the area fraction of the precipitates. Therefore, the yield strength produced by the precipitates, $∆\sigma_{p}$, is calculated to be 73 MPa of the extruded MB alloy and 89 MPa of the extruded MBA alloy.

Here, the contribution of solid solution strengthening could be negligible because the numerous Mg_3_Bi_2_ precipitates have consumed many Bi atoms. Consequently, the tensile yield strength of the experiment alloys can be analyzed approximately using following equation [60]:

(8) $$\sigma_{0.2-cal}={\;\sigma }_{0\;}+\Delta \sigma_{gb}+{\Delta \sigma }_{d}+{\Delta \sigma }_{p}$$

where *σ*_0.2*-cal*_ expresses the calculated tensile yield strength, and *σ*_0_ (~11 MPa [50]) is the lattice friction stress for Mg. Therefore, the calculated values of *σ*_0_, Δ*σ_gb_*, Δ*σ_d_*, Δ*σ_p_*, and *σ*_0.2*-cal*_ are listed in Table 2. The calculated *σ*_0.2-*cal*_ is almost in agreement with the tested *σ*_0.2_ of 256 ± 5.0 MPa for the extruded MB alloy, and 304 ± 2.0 MPa for the extruded MBA alloy.

### 4.3. Strain Hardening Capacity

The strain hardening of alloys is believed to be one of the important deformation behaviors, including Mg alloy [31,35,36,37]. It closely relates to the accumulation and annihilation of dislocations during plastic deformation of alloys. Thus, the strain hardening of alloys is produced by dislocation accumulation, whereas dislocation annihilation brings about the softening, resulting in the decrease in the strain hardening. The factors related to dislocation density inside the grains, including secondary phases, grain size, twinning, and texture etc., might affect the strain hardening capacity of the alloys [32,35,61,62,63]. To consider the strengthening behavior affected by grain size and dislocation after yield, the relation of the dislocation density ρ and net flow stress $\left(\sigma -\sigma_{0.2}\right)$ can be expressed as follows [61]:

(9) $$\rho^{1/2}\propto \;\sigma_{D}\approx \left(\sigma -\sigma_{0.2}\right)$$

where *σ_D_* is the strength contribution by dislocation density, *σ_0.2_* is the yield stress. Thus, the well-known Taylor dislocation contribution to strength, can be given [61]:

(10) $$\sigma_{D}=M\alpha Gb\rho^{1/2}$$

where *G* means shear modulus, *M* indicates the Taylor factor, *α* is a constant, and *b* expresses Burgers vector.

The dislocation storage rate can be given as following equation, which is based on the Kocks strain hardening model [31,36]:

(11) $$\frac{d\rho }{d\gamma }=A+B\rho^{1/2}-C\rho$$

where $A={\left(bd\right)}^{-1}$ means the coefficient of dislocation storage, which is related to grain size or particle space, and *B* indicates the coefficient of dislocation storage resulted from the interaction between forest dislocations. Both the *A* and $B\rho^{1/2}$ terms express the dislocation accumulation, resulting in strain hardening behavior. *C* is the coefficient of dislocation annihilation arisen from dislocation cross-slip, leading to softening of alloy due to dynamic recovery.

In addition, the relation between strain hardening rate θ and dislocation storage is described as follows:

(12) $$\theta =\frac{d\tau }{d\gamma }=\frac{\alpha \mu b}{2\rho^{1/2}}\frac{d\rho }{d\gamma }$$

**Figure 14:**
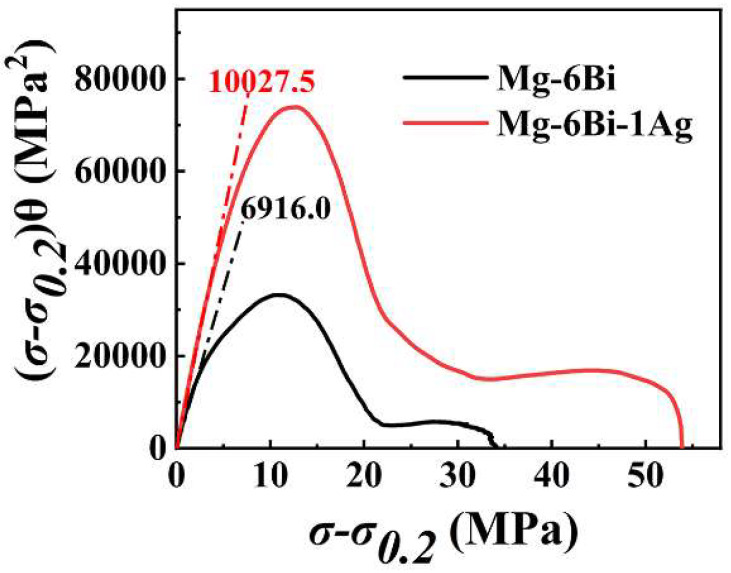
(*σ-σ*_0.2_)*θ* vs. (*σ-σ*_0.2_) curves for the extruded (experiment) alloys.

Considering Equations (9) and (12), it can be understood that the (*σ-σ*_0.2_)*θ* vs. (*σ-σ*_0.2_) is equivalent to $\frac{d\rho }{d\gamma }$ (dislocation storage) vs. $\rho^{1/2}$ (dislocation density). Therefore, the curve of (*σ-σ*_0.2_)*θ* vs. (*σ-σ*_0.2_) can reflect the relationship between $\frac{d\rho }{d\gamma }$ and $\rho^{1/2}$. According to Figure 10b, the curves of (*σ-σ*_0.2_)*θ* vs. (*σ-σ*_0.2_) are obtained, as shown in Figure 14. The dashed lines in Figure 14 are the fitted lines passing through the origin, which indicate the slope of the first part of these curves at low stress. From Figure 14, it is observed that the slope value of the curve is greater for the extruded MBA alloy than that for the extruded MB alloy. This result demonstrates that the MBA alloy possesses greater dislocation storage ability during the deformation process, showing a large strain hardening capacity. Furthermore, the parameters *A*, *B*, and *C* described in Equation (11) can be obtained by fitting the curves shown in Figure 14. Thus, the values of these parameters can be seen in Table 3. This demonstrates that *A*, *B*, and *C* parameters of the extruded MBA are higher than those of the extruded MB alloy. Using the values of A, B, and C, it is implied that Ag can increase the storage capacity of dislocations of the alloy due to higher A and B values, improving the strain hardening capacity of the MBA alloy. However, the higher *C* value for the extruded MBA alloy infers that the dislocation annihilation rate increases due to dislocation cross-slip, leading to increase dynamic recovery rate. These results are consistent with those given in Figure 10.

Many previous literatures have reported the effect of grain size on strain-hardening behaviors [34,35,62,63,64,65]. The fine grain size can reduce the dislocation storage in alloys, slowing down the strain hardening rate. However, the two alloys exhibit the almost same average DRXed grain size (*d_DRXed_*), as presented in Figure 4. Meanwhile, the unDRXed grain size (42.3 µm) of the extruded MB alloy is larger compared to that (17.7 µm) of the extruded MBA alloy. However, the results obtained from Equation (11) illustrate that the dislocation storage ability and strain-hardening capacity for the extruded MB alloy with large grain size is lower in comparison to MBA alloys during uniaxial tensile deformation. This means that the grain size is not the only factor that affects the dislocation storage ability for the present extruded alloy. However, Figure 10b indicates that the slope of the curve at stage III deformation for the extruded MB alloy exhibits a smaller value, which represents the lower dynamic recovery rate. It should be mainly ascribed to the larger unDRXed grain size and higher content of the unDRXed grains. Zhang et al. [66] reported that dislocation density in large unDRXed grain size increases with the tensile strain increasing, which is on account of the presence of more free-slipping paths and fewer obstacles to inhibiting dislocation slipping. Consequently, the dynamic recovery rate is weakened for the extruded MB alloy.

As reported in previous literature [32,35,36,61], it can be understood that the extruded MBA alloy with fine grain size demonstrates a slightly higher dynamic recovery rate at stage III compared to the extruded MB alloy. This is attributed to the dislocation saturation easily occurring and the dislocation easily annihilating at the grain boundary in fine grains on account of the high no-equilibrium energy in the fine-grain alloy [67].

As indicated in Figure 10b, with the increase of (*σ-σ*_0.2_), the extruded MBA alloy exhibits that the strain hardening rate declines slightly faster than the extruded MB alloy in stage IV deformation and the longer stage IV deformation. It has been reported [38] that at the large strain region, stage IV appeared in the strain-hardening behavior should be relevant to the accumulation of dislocation debris, which causes dislocation rearrangements to the formation of dislocation tangles or cell walls. The higher dislocation storage can offer a greater driving force for the rearrangement of dislocation debris to form the cell substructure or even the annihilation of dislocation. As stated above, the extruded MBA alloy exhibits higher dislocation storage compared to MB alloy, which leads to providing a greater dynamic recovery rate at stage IV due to easy rearrangement or annihilation of dislocation [33,38].

In addition to the grain size, the precipitates also can affect the strain-hardening behavior as an obstacle preventing dislocation motion. Therefore, the strain-hardening rate of the alloy can increase with the volume of precipitates increasing. On the other hand, the precipitates can impede the dislocation moving, which can slow down the strain softening rate due to the reduction of the dislocation annihilation rate [35]. The present results indicate that more area fraction of precipitates in MBA alloy should favor improvement of strain-hardening capacity due to the high storage of dislocation indicated in results obtained by Equation (11), although fine grain size slightly increases the dynamic recovery rate. As reported by Habibnejad-Korayem [68], the finer precipitates in the Mg matrix could trap the dislocations during the deformation process, resulting in easily forming the dislocation networks and loops. Therefore, the back stress could be produced by the dislocation loops, which make the dislocations movement more difficult. As a result, the annihilation rate of dislocation forests and dislocation cells can slow down, and the stage of dynamic recovery could maintain longer in MBA alloy. It is also favorable to improve the strain-hardening capacity.

Nevertheless, to further comprehend the effect of precipitates on strain-hardening behavior, more research needs to be carried out in the future because the comprehensive relation between them is complex, as stated by Cheng [69].

## 5. Conclusions

Based on the investigation of the microstructure and mechanical behaviors of experiment alloys, the main conclusions are as follows:

(1) The microstructures of two experiment alloys exhibit the bimodal structure consisting of the large unDRXed grain size and fine DRXed grain size after extrusion at 300 °C and the die-exit speed of 2 m/min with the extrusion ratio of 25:1. Ag addition can promote the DRX, leading to increasing content of DRXed grains and decreasing the average grain size of the alloy.

(2) Ag obviously accelerates the dynamic precipitation of Mg_3_Bi_2_ phases in extruded Mg–6Bi–Ag alloy and increases the content of dynamic Mg_3_Bi_2_ precipitates during extrusion. The nanosized precipitates can form in the extruded MBA alloy within grains with many dislocations.

(3) The Ag addition can increase the strength of the extruded alloy. The results show an ultimate tensile strength of 332 ± 4.8 MPa and yield strength of 304 ± 2.0 MPa for the extruded MBA alloy. The yield strength is increased by 19%. The fracture elongations indicate 11.0 ± 1.7% of the extruded MBA alloy and 11.9 ± 0.9% of the extruded MB alloy.

(4) The strain-hardening capacity of the extruded MBA alloy can be increased by the addition of Ag. However, the strain-hardening rate for the extruded MBA alloy at stage III and stage IV decreases slightly faster compared to MB alloy, which is on account of fine grain size promoting dynamic recovery.

## Figures and Tables

**Table 1:** Mechanical properties of experiment alloys.

Alloy	*σ*_0.2_ (MPa)	*σ*_UTS_ (MPa)	EL (%)	*σ*_0.2-T_ (MPa)	*σ*_UTS-T_ (MPa)	*e*_T_ (%)
MB	256 ± 5.0	276 ± 2.3	11.9 ± 0.9	258 ± 5.6	295 ± 4.0	11.3 ± 0.9
MBA	304 ± 2.0	332 ± 4.8	11.0 ± 1.7	309 ± 1.9	364 ± 1.6	10.4 ± 1.5

Note: *σ*_0.2_, *σ*_UTS_ and EL: engineering strength and tensile elongation, *σ*_0.2-T_, *σ*_UTS-T_ and *e*_T_: true strength, and true tensile elongation.

**Table 2:** The individual strengthening contribution and *σ_*0.2*-cal_* of extruded alloys.

Alloy	*σ*_0_ (MPa)	Δ*σ_gb_* (MPa)	Δ*σ_d_* (MPa)	Δ*σ_p_* (MPa)	*σ*_0.2*-cal*_ (MPa)
MB	11	92	79	73	255
MBA	11	116	87	89	303

**Table 3:** Fitted parameters *A*, *B*, and *C* shown in Equation (11).

Alloy	*A* (MPa)^2^	*B* (MPa)	*C*	*R* ^2^
MB	174.6	5586.5	340.3	0.989
MBA	430.0	11,711.4	459.6	0.999

## Data Availability

Data are contained within the article.
